# Comparative In Vitro Biocompatibility Study of the Two Orthodontic Bonding Materials of Different Types

**DOI:** 10.3390/polym14224998

**Published:** 2022-11-18

**Authors:** Predrag Janošević, Sanja Stojanović, Ivana Stojanović, Mirjana Janošević, Stevo Najman

**Affiliations:** 1 Department of Orthodontics, Faculty of Medicine, University of Niš, 18000 Niš, Serbia; 2 Department of Biology and Human Genetics, Faculty of Medicine, University of Niš, 18000 Niš, Serbia; 3 Department for Cell and Tissue Engineering, Faculty of Medicine, University of Niš, 18000 Niš, Serbia; 4 Department of Biochemistry, Faculty of Medicine, University of Niš, 18000 Niš, Serbia

**Keywords:** in vitro, biocompatibility, orthodontic bonding materials, fibroblasts, macrophages

## Abstract

In the present study, the in vitro biocompatibility and cell response to two commonly used orthodontic bonding materials of different types, one self-curing and one light-curing, were examined and compared in indirect and direct cell culture systems. The study was conducted on fibroblasts and macrophages as in vitro models to study the biocompatibility of dental materials. Differences were found between the light- and self-curing material in cytotoxicity and effects on fibroblasts’ proliferation in indirect cell culture systems as well as in macrophages response in vitro in both direct and indirect cell culture systems. Based on the obtained results, we can conclude that the self-curing material is generally more cytotoxic for fibroblasts compared to the light-curing, while macrophages’ response to these materials was dependent on the macrophages’ state and differed between the examined materials. This indicates that more attention should be paid when choosing and applying these materials in practice due to their toxicity to cells. Prior to their use, all aspects should be considered regarding the patient’s conditions, associated problems, microenvironment in the oral cavity, etc. Further studies on in vivo models should be conducted to fully understand the potential long-term effects of the use of mentioned materials in orthodontics.

## 1. Introduction

Determining the biocompatibility of the orthodontic bonding materials used for gluing the braces in fixed orthodontic appliance therapy is very important since they are located very close to periodontal tissue and the alveolar bone. The substances released from the bonding materials could cause inflammatory or even necrotic changes in the mentioned tissue, which could induce harmful effects on the patients. Orthodontic bonding materials can cause changes in the tissue either due to direct contact or due to the release of soluble components in the gingival sulcus fluid and saliva [1].

The literature data indicate that considerable amounts of monomers and short chains of polymers remain unbound within the structure of the adhesive mass and that their release is the main cause of cytotoxicity in a short time interval after bonding [2,3]. Additionally, dental composites have the ability to absorb a certain amount of water, which leads to the additional displacement of residual monomer and the intensification of toxic effects [4]. In vivo studies have shown that there is an inflammatory reaction, especially to the liquid component of the adhesive material in all of the tested animals [5]. Some studies have revealed that there are ultrastructural changes in the epithelium due to the penetration of the primer from the material [6]. Additionally, there are studies reporting the genotoxic effect of the mentioned materials [7,8], which also raises concerns about the safety of these materials. There are different types of orthodontic adhesives. They can be broadly classified into two main categories: diacrylates (composite resin) and glass ionomer cements (GICs), which may be set by either chemical reaction or by photopolymerization [9]. In adhesives that are set by chemical reaction, autopolymerization occurs when all the components are mixed with each other, and these adhesives are usually called self-curing, while those that are set by photopolymerization after mixing the components are called light-curing. The second proved to be potentially safer since polymerization occurs faster and fewer residual monomers exist.

Some tests performed on fibroblasts have shown that self-curing materials are more toxic compared to light-curing materials [10]. Studies reporting on the toxic effects of these materials appeared a long time ago, but these materials are still used, although with a different composition that changes rapidly, with the aim to find components that are biocompatible and retain all the necessary properties. The largest number of studies refers to the physical properties of these materials, while significantly fewer studies are dedicated to their biocompatibility and potential side effects. Orthodontic bonding materials may also be used for other indications in dentistry, such as for splinting and bonding the retainers [11], which raises concerns about the cytotoxicity of these materials taking into account how long lingual fixed retainers stay in the mouth.

Although orthodontic bonding materials are widely used despite their potential side effects, there is a lack of studies dealing with the biocompatibility of orthodontic bonding materials and experimental data providing comparable results for various types of materials at cellular levels. The aim of this study was to examine and compare the in vitro biocompatibility and cell response to the two commonly used orthodontic bonding materials, one self-curing and one light-curing, in indirect and direct cell culture system on fibroblasts and macrophages as models to study biocompatibility of dental materials. The first hypothesis tested in the present study was that there is a significant difference in the cytotoxicity of the tested materials. Since increased gingival inflammation is a common side effect in patients with fixed orthodontic appliances [12], the aim of this study was to analyze the impact of orthodontic bonding materials in the induction of inflammatory response using in vitro direct culture system with macrophages. Bearing in mind that in the moment of the placing orthodontic appliance patient’s gingiva may be sub-clinically inflamed or the patient may have low-grade chronic inflammation, the aim of this study was to also examine whether orthodontic bonding materials can intensify existing low-grade inflammation by testing these materials on macrophages already stimulated with an inflammatory agent in a direct culture system in vitro. The second hypothesis tested was that there is a significant difference in the macrophages’ response to the tested materials depending on the macrophages’ functional state.

## 2. Materials and Methods

### 2.1. Materials

Non-mix self-cure acrylic (labeled SC) by DENTAURUM GmbH & Co. KG (Ispringen, Germany) and Transbond™ XT Light Cure (labeled LC) by 3M (St. Paul, MN, USA), the two commercially available orthodontic bonding materials of a different type, were tested and compared in this study. The details about the tested materials and their composition are given in Table 1. The materials were tested in a polymerized form, after mixing the appropriate components (activator and adhesive in the case of self-curing material; primer and adhesive in the case of light-curing material) and polymerization, according to the manufacturers’ instructions and in the way it is prepared and used in clinical practice.

### 2.2. Cell Lines

The in vitro biocompatibility testing of the materials was performed on two commercial cell lines, the L929 (mouse fibroblasts) and RAW 264.7 (mouse macrophages) cell lines, both obtained from American Type Culture Collection (ATCC, Manassas, VA, USA). The L929 cell line was taken for indirect culture system testing on fibroblasts, as it is the most commonly used model for testing dental materials on fibroblasts. The RAW 264.7 cell line is a macrophage line used for phagocytosis assay and immunomodulatory activity testing of materials. The cells of both cell lines were cultured in Dulbecco’s Modified Eagle Medium with 1 g⁄L glucose (DMEM) supplemented with 2 mM stable glutamine, 1% antibiotic-antimycotic solution, and 10% fetal bovine serum (complete DMEM), all purchased from Biological Industries (BioInd, Kibbutz Beit-Haemek, Israel), in standard cell culture conditions (humidified atmosphere, 5% CO_2_, 37 °C).

### 2.3. Preparation of Materials for Testing

The preparation of the materials before cell testing was performed according to the manufacturer’s recommendations and in the same way as in clinical practice. The plates of the tested materials with approximate dimensions of 8 × 5 × 1 mm were made in sterile plates following the procedures prescribed for their clinical use. In the case of the light-curing material, the components are applied to the dish, and then it is light-cured. In the case of self-curing materials, the components are gently mixed and left to polymerize after rapid application to the dish. After the completion of self- or light-polymerization, depending on the type of test material, the sample plates were further used for testing. Before testing the materials on the L929 cells, extracts of the materials in their polymerized form were made. The extraction was performed at 37 °C in a DMEM medium to which an antibiotic–antimycotic solution was added. The material/medium ratio was 0.2 g per 5 mL of medium. One-day, three-day, and seven-day extracts of both materials were made. After the extraction period was completed, fetal bovine serum (final concentration 10%) and stable glutamine (2 mM) were added to the extracts. The extracts were diluted to certain final concentrations with complete DMEM. The final examined extract dilutions were: 100 (undiluted), 50, 25, 10, and 5%. For the examination of the materials on the RAW 264.7 macrophages, the plates of polymerized SC and LC were crushed with a mortar and pestle to a coarse powder consistency and examined in the form of a suspension in a direct contact culture system.

### 2.4. Indirect Contact Assay on Fibroblasts

The effect of the material extracts on the viability and proliferation of L929 fibroblasts was examined using an indirect contact cell culture system. The cells were detached using Trypsin-EDTA (Biological Industries, Israel), centrifuged, washed, counted, and seeded in 96-well tissue culture plates (Greiner Bio-One, Kremsmünster Austria). For the viability assay, 2 × 10^4^ cells per well were seeded, while for the proliferation assay, 5 × 10^3^ cells per well were seeded. After 24 h of cultivation, in both assays, the material extracts were added in the above-mentioned dilutions, as well as complete DMEM as a control. The cells were incubated with the material extracts as well as a control medium for the next 24 h in the viability assay or 72 h in the proliferation assay, under standard cell culture conditions. After the incubation period ended, an MTT test was performed in both assays.

### 2.5. Testing of Materials on Macrophages’ Metabolic Activity

The effect of the material extracts, as well as the suspension of material particles, on the metabolic activity of RAW 264.7 macrophages in the presence and absence of low concentration of an inflammatory agent, was tested as well. The cells were detached using a cell scraper (Sarstedt, Nümbrecht, Germany), centrifuged, washed, counted, and seeded in 96-well culture plates. For this assay, 2 × 10^4^ cells per well were seeded, and after 24 h of cultivation, the material extracts (concentrations 100, 25, and 5%) and the suspension of the material particles (concentrations 100 and 20 µg/mL) with and without the addition of 1 ng/mL of lipopolysaccharide (LPS, 0111: B4 *Escherichia coli*, Sigma-Aldrich, St. Louis, MO, USA) were added to the cells. As controls, complete DMEM (unstimulated macrophages), and LPS (LPS-stimulated macrophages) were used. The cells were incubated with material extracts and suspension, as well as control media, for the next 24 h, under standard cell culture conditions. After the incubation period ended, an MTT test was performed.

### 2.6. MTT Test

The MTT test is widely used for the assessment of cell proliferation and is based on the reduction of 3-(4,5-dimethylthiazol-2-yl)-2,5-diphenyl-2H-tetrazolium bromide (MTT) by the mitochondrial dehydrogenases of the living cells, resulting in the formation of formazan crystals that corresponds with the number of cells. The test was performed according to the already used protocol in our laboratory [13]. The cells were washed with phosphate-buffered saline, and then 100 μL of the MTT solution at a concentration of 1 mg/mL (MTT, Carl Roth, Karlsruhe, Germany) per well was added to the cells. The cells were incubated with an MTT solution for the next three hours, followed by the dissolution of the formed formazan dissolution with 100 µL of 2-propanol per well. The absorbance of the dissolved formazan was measured on a Multiskan Ascent Photometric plate reader (ThermoLab Systems, Helsinki, Finland) at a wavelength of 540 nm with a correction wavelength of 650 nm. The mean absorbance values were calculated for each tested sample, as well as for the controls. The cell viability and proliferation rate were calculated according to the following formula: % cell viability/proliferation = (absorbance value of treated cells/absorbance value of control cells) × 100.

### 2.7. Macrophages’ Response to Materials’ Particles

To study the macrophage response to the materials, RAW 264.7 cells were seeded at a density of 2 × 10^5^ cells per well (in 2 mL of complete DMEM) in a sterile 12-well culture plate (Greiner Bio-One, Kremsmünster Austria). After 24 h of cultivation under standard cell culture conditions, the medium was removed, and the suspension of SC or LC material particles, made with complete DMEM, with and without the addition of 1 ng/mL of LPS, was added to the cells. Two concentrations of materials’ suspensions, 100 and 20 µg/mL, each in three replicates, were tested. Phagocytosis of the material particles was observed after 24 h incubation with material suspensions on an inverted light microscope Axio Observer Z1 (Carl Zeiss, Oberkochen, Germany) under phase contrast. The images were acquired using the camera AxioCam HR in the software ZEN 2 blue edition (Carl Zeiss, Oberkochen, Germany). The macrophage inflammatory-related secretion products were measured in media 24 h after exposure to the materials.

### 2.8. Enzyme-Linked Immunosorbent Assays (ELISA)

The secretion of TNF-α, a pro-inflammatory cytokine, by the RAW 264.7 cells cultured for 24 h with the material suspension, as well as controls, was measured in the cell supernatant using an enzyme-linked immunosorbent assay (ELISA) using Mouse TNF-alpha Quantikine ELISA Kit (MTA00B, RnD systems, Minneapolis, MN, USA). The assay was performed according to the manufacturer’s instructions. The values are expressed as pg of TNF-α per mL.

### 2.9. Nitric Oxide Determination

Nitric oxide (NO) production was indirectly estimated using the spectrophotometric measurement of its stable decomposition product nitrite in the media of RAW 264.7 cells exposed for 24 h to the material suspension and control media, using Griess reagent according to the already used protocol in our laboratory [13]. Briefly, 100 μL of cell supernatant was added to an equal volume of Griess reagent and incubated for 10 min at RT. The absorbance was measured at 540 nm. The nitrite concentration in the medium was calculated from the sodium nitrite (NaNO_2_) standard curve.

### 2.10. Statistical Analysis

All of the results were statistically processed, and for all samples, the mean values were calculated. The results of the viability and proliferation assays are presented as a percentage with relative standard deviation (RSD), while the results of the macrophage metabolic activity and the secretion product measurements are presented as mean values with standard deviation (SD). Statistically significant differences between the samples were analyzed using a Student’s *t*-test of independent samples, Mann–Whitney U-test, and one-way analysis of variances (ANOVA). The values of *p* ≤ 0.05 were considered significant.

## 3. Results

### 3.1. Effect of Materials’ Extracts on Viability and Proliferation of Fibroblasts

**Figure 1:**
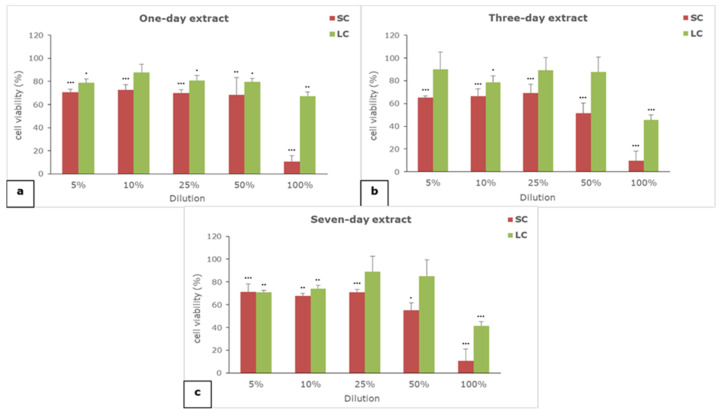
The effect of (**a**) one-, (**b**) three-, and (**c**) seven-day extracts of materials on the viability of L929 cells; SC—self-curing material; LC—light-curing material; (*) *p* < 0.05, (**) *p* < 0.01, (***) *p* < 0.001.

The results of the effect of one-, three-, and seven-day extracts of SC and LC material on cell viability are given in Figure 1.

All of the extracts of both of the examined materials showed concentration-dependent cytotoxicity. The self-curing material extracts are generally more cytotoxic than the light-curing material extracts, regardless of the extraction period and the tested concentration. All of the undiluted SC extracts (100%) proved to be seriously cytotoxic, while moderate to slight cytotoxicity was noticed for the diluted SC extracts. On the other hand, only undiluted (100%) three- and seven-day LC extracts exerted moderate cytotoxicity, while all other dilutions were not cytotoxic or exerted slight cytotoxicity.

**Figure 2:**
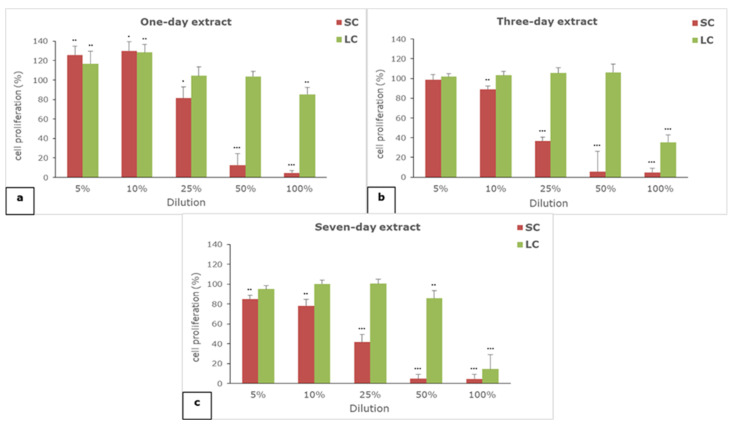
The effect of (**a**) one-, (**b**) three-, and (**c**) seven-day extracts of materials on the proliferation of L929 cells; SC—self-curing material; LC—light-curing material; (*) *p* < 0.05, (**) *p* < 0.01, (***) *p* < 0.001.

The results of the effect of one-, three-, and seven-day extracts of SC and LC material on cell proliferation are given in Figure 2.

All of the extracts of both of the examined materials showed concentration-dependent effects on cell proliferation. The self-curing material extracts generally have a greater anti-proliferative effect than the light-curing material extracts. All of the extracts of the SC material exerted serious anti-proliferative effects in concentrations of 50 and 100%, while in the case of LC extracts, only 100% seven-day extract showed such an effect. The moderate anti-proliferative effect was observed for three- and seven-day SC extracts in concentration of 25% and 100% of three-day LC extract. In lower examined concentrations (5 and 10%), only a slight anti-proliferative effect was noticed for the seven-day SC extract, while the proliferation of fibroblasts was not reduced in the case of other extracts, or it was slightly increased in the case of the one-day SC and LC extracts.

### 3.2. The Effect on Metabolic Activity of Macrophages

**Figure 3:**
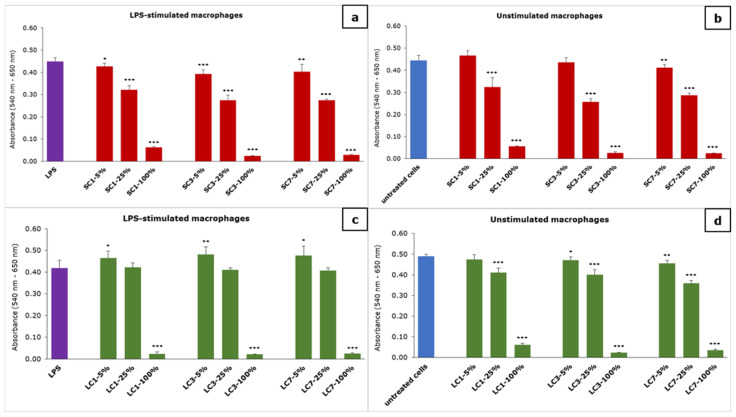
The effect of the material extracts on metabolic activity of macrophages: (**a**) effect of SC-material’s extract on LPS-stimulated macrophages, (**b**) effect of SC-material’s extract on unstimulated macrophages, (**c**) effect of LC-material’s extract on LPS-stimulated macrophages and (**d**) effect of LC-material’s extract on unstimulated macrophages; results are presented as mean ± SD (*) *p* < 0.05, (**) *p* < 0.01, (***) *p* < 0.001; SC—self-curing; LC—light-curing material.

The results of the effect of the material extracts on the metabolic activity of macrophages are presented in Figure 3.

The concentration-dependent effect of the material extracts on the metabolic activity of macrophages, measured using the MTT test, was noticed. In the case of the SC-material extract, a significant reduction in macrophage metabolic activity was noticed for all of the examined dilutions, while it was not reduced in the case of 5 and 25% of one- and three-day extract on unstimulated macrophages. In the case of the LC-material extract, a significant reduction in LPS-stimulated macrophages was noticed for only the undiluted extracts (100%), while a 5% dilution of all extracts significantly increased the metabolic activity. When the LC-material extract was examined on unstimulated macrophages, a reduction in metabolic activity was noticed for all dilutions except for 5% of the one-day extract.

**Figure 4:**
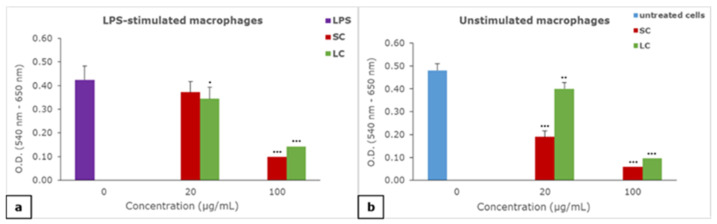
The effect of suspension of material particles on the metabolic activity of macrophages: (**a**) effect of materials on LPS-stimulated macrophages, (**b**) effect of materials on unstimulated macrophages; results are presented as mean ± SD (*) *p* < 0.05, (**) *p* < 0.01, (***) *p* < 0.001; SC—self-curing; LC—light-curing material.

The presence of 100 µg/mL of both the SC and LC material suspension significantly decreased the metabolic activity of both LPS-stimulated (Figure 4a) and unstimulated macrophages (Figure 4b) with a more pronounced effect of the SC material. Both SC and LC significantly decreased the metabolic activity of unstimulated macrophages at a concentration of 20 µg/mL, with a more pronounced effect of SC, while a significant change in the metabolic activity of LPS-stimulated macrophages was noticed in the case of LC at the same concentration.

### 3.3. Phagocytosis and Effects on Macrophages’ Response

**Figure 5:**
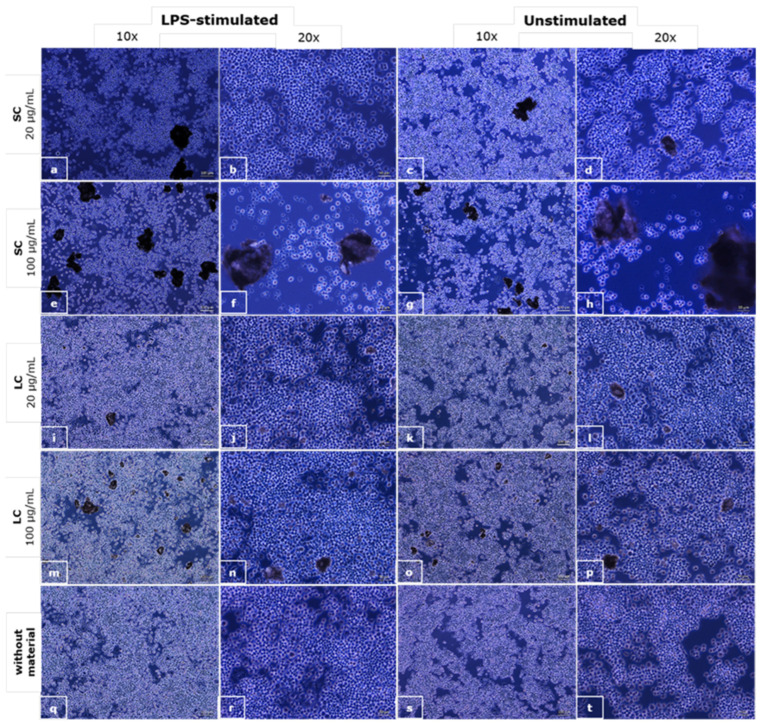
The microscopic appearance of the macrophages incubated with a suspension of SC (**a**–**h**) and LC (**i**–**p**) material particles and control media (**q**–**t**); phase contrast, objective magnifications 10× (scale bar shows 100 µm) and 20× (scale bar shows 50 µm).

In Figure 5, the appearance of the RAW 264.7 macrophages exposed to the material particles is shown.

The influence of the particles of the materials in suspension on macrophage morphology was observed as well. The dead cells can be seen in the case of the SC material at a concentration of 100 µg/mL regardless of the macrophage stimulation. Changes in cell morphology can be seen in all LPS-stimulated cultures, with and without materials’ particles, where cells were bigger and wider, and cells with vesicles can be seen. Signs of phagocytosis are observed in the interaction of smaller particles with macrophages.

We used LPS-stimulated macrophages as a positive control for phagocytosis, as LPS stimulates the phagocytic activity of the macrophages. In the culture of macrophages exposed to the suspension of SC material particles at a concentration of 100 µg/mL, there are no visible signs of phagocytosis, but a reduction in the number of cells compared to the control and apoptotic cells can be seen. In the culture of the macrophages exposed to a suspension of SC material at a concentration of 20 µg/mL, there is a slight reduction in the number of cells, but also, there are signs of phagocytosis of the material particles. In the culture of the macrophages exposed to suspensions of LC material in concentrations of 100 and 20 µg/mL, pronounced phagocytosis of the particles can be observed, while there are no signs of apoptosis.

The results of the macrophage response and the secretion of inflammatory-related products in the presence of the material particles are presented in Table 2 and Table 3.

When the suspension of the materials was examined on unstimulated macrophages, significantly lower concentrations of TNF-α were noticed in the case of SC material in both of the examined concentrations, while it was significantly higher in the case of LC material in both examined concentrations with pronounced effects of higher examined concentrations, compared to unstimulated macrophages (Table 2). When the suspension of the materials was examined on LPS-stimulated macrophages, a significantly lower concentration of TNF-α was noticed in all examined cases except in the case of LC material concentration 100 µg/mL when it was higher compared to LPS-stimulated macrophages (Table 2).

There were no changes in the concentration of the nitrites produced by unstimulated macrophages without and with the suspension of examined materials (Table 3). Significantly lower concentrations of nitrites were detected when LPS-stimulated macrophages were incubated with both SC and LC materials’ suspension at concentrations of 20 µg/mL and with the LC material suspension at a concentration of 100 µg/mL, while a significantly higher concentration of nitrites was measured in the case of the SC material suspension at a concentration of 100 µg/mL.

## 4. Discussion

Both hypotheses set up in this study were accepted. The results show that there is a statistically significant effect of the material extracts, especially the SC material, on the viability and proliferation of fibroblasts and that this effect is concentration- and extraction period-dependent.

The release of residual monomers is thought to be the main cause of the cytotoxicity of the bonding materials. It has been shown that approximately 5 to 10% of the residual monomer is usually poured into the solution [2]. Bisphenol A-diglycidyl dimethacrylate (Bis-GMA), the most commonly used derivative of Bisphenol A in epoxy-resin-based dental composites, is a monomer with methyl methacrylate groups bound to hydroxyl groups of Bisphenol A [14]. It has been shown that varying amounts of Bisphenol A could be released from different dental materials in various conditions, measured both in vitro and in vivo, which may have a negative impact on patient health [15,16,17]. Bis-GMA was shown to be the main monomer released from dental composites and potentially the most toxic component among the dimethacrylate derivatives [18,19,20]. Some studies have shown that the degradation derivatives of dental composites can cause a similar toxic effect as monomers [4]. While some studies reported that the material’s incubation period of 7 days at a temperature of 37 °C in cell culture is sufficient to indicate the maximum cytotoxic potential of the mentioned materials [4], other studies indicate that the highest amount of Bis-GMA is released in the first 30 min after polymerization [21]. In addition to Bis-GMA, other important dental dimethacrylate monomers include Bis-EMA (bisphenol A ethoxylate dimethacrylate), UDMA (urethane-dimethacrylate monomer), and TEGDMA (triethylene glycol dimethacrylate), which are usually used in mixtures of different ratios and then copolymerized [22]. It has been reported that the properties of dental materials result from the interaction of several structural factors, such as the monomer chemical structure, polymer network molecular structure (crosslink density, which relates to the chemical crosslink density, physical crosslink density, and degree of conversion), as well as morphology [22]. The copolymerization of basic monomers, such as Bis-GMA and UDMA, with reactive diluents, such as TEGDMA, results in better physicochemical and mechanical properties of dental materials [22].

The bond of composite orthodontic adhesive materials usually consists of two main components, two monomers: bis-GMA and TEGDMA. TEGDMA is a major component of dentin-bonding agents and dental resin composites [23,24] -and is used as an integral part of the bond, while 2-hydroxyethyl methacrylate TEGDMA (HEMA/TEGDMA) is an integral part of the base mass of the composite. Animal studies have indicated that there is a biodegradation concern for HEMA/TEGDMA. It was shown that both molecules are easily released from the material after application [25,26,27]. TEGDMA, Bis-GMA, and urethane dimethacrylate (UDMA) are hydrophobic monomers, but they are often found in composites bound to HEMA, which increases the hydrophilic characteristics of the material. Some studies have shown that the addition of HEMA to the polymer matrix improves the mechanical properties of the composite made of Bis-GMA and UDMA, which may be related to the improved mobility of the bis-GMA and UDMA monomers [28].

Dimethacrylate polymers are generally non-toxic, but due to incomplete conversion and the chemical degradation of dimethacrylate polymer networks, low molecular weight substances, dimethacrylate monomers, can be released and eluted by saliva and may induce a variety of adverse biological effects, mainly toxicity, to cause cell damage [29,30,31] and other harmful effects on oral tissues [22]. It has been shown that TEGDMA is less toxic than Bis-GMA and UDMA [22].

TEGDMA monomers are reported to induce dental pulp inflammation and necrosis [23]. Orthodontic bonding materials are located near the gingival sulcus for a long time, achieving direct and long-term contact with the epithelium that does not keratinize, so it is a weaker barrier for the penetration of the mentioned molecules. Because of all the above, there is a tendency nowadays to develop BIS-GMA-free materials to reduce the toxic effect of orthodontic bonding materials [17,32]. The main dimethacrylate monomer of the tested materials in the present study is BIS-GMA, which means that toxic effects can be attributed to this component of the tested materials. However, the LC material proved to be generally less cytotoxic, which means that polymerization was much more successful in the case of the LC material or the presence of other components such as TEGMA influenced better properties of the LC material.

The results obtained in the conducted study show that there are differences in the effect of the material extracts related to the extraction period, which indicates that with time, the materials release potentially toxic substances. These differences are more pronounced when the effect on the proliferation of fibroblasts was examined. The obtained results partially coincide with the results of other studies that reported a mild cytotoxic effect of the LC orthodontic materials only on the seventh day, which they explained by the possibility that the materials then additionally absorbed water, which is the condition that causes the release of monomer [3]. Authors of other studies came to the conclusion that the cytotoxicity of all tested materials decreases from the seventh day of pre-incubation [33,34]. Variable trends of cytotoxicity throughout the entire duration of the experiment were also noticed by other authors [35,36]. In one study that examined LC materials, it was concluded that four out of five examined SP composites are safe for use with one, Transbond XT, which is also examined in the present study, reported to be cytotoxic for L929 cells after 24 h [37]. In another study where three different LC materials were tested, including Transbond XT, authors reported cytotoxic effects on L929 fibroblasts also after one day [38].

The results of the present study show that the extracts of the LC material are cytotoxic and are anti-proliferative at a concentration of 100%, although significantly less than the SC material extract. The reason for the greater cytotoxicity and anti-proliferative effect of the extracts of SC material can be partly the technique of preparing SC material samples for application. The polymerization of the SC material requires the mixing of the bond and the basic components of the composite, which results in a slightly larger amount of bond used in the production of the material’s samples for the experiment than in the production of the samples of the LC material. Excess applied bond easily leaks from the polymerized material and dissolves in saliva and gingival sulcus fluid. The obtained results match the results obtained by authors in other studies [3,21].

In the conducted study, we made dilutions of material extracts since the elution and dilution of these materials with saliva inevitably occur in the oral cavity. However, saliva does not remain in the oral cavity for long but is directed to further parts of the alimentary tract by swallowing. Considering the proximity of the adhesive material to the gingival sulcus and the very small amount of the fluid there, it may be more appropriate to pay attention only to the 50% and 100% extract concentrations of the tested materials when discussing their toxic effect on the gingival tissue. Since the obtained results indicate a significant decrease in the viability and proliferation of fibroblasts exposed to these concentrations of extracts, we can conclude that SC and LC materials represent a risk factor for the occurrence of gingival irritation and may lead to gingival inflammation.

According to our knowledge, there are no studies in the literature that dealt with the influence of orthodontic adhesive materials for brackets’ gluing on macrophages. There is also no research into the ability of macrophages to phagocytose small particles of these materials. The obtained results indicate that there is a pronounced toxic effect of SC material suspension at a concentration of 100 µg/mL on macrophages that is visible through apoptotic cell appearance in Figure 5e–h. This effect is not seen when the LC suspension was examined on macrophages. However, signs of phagocytosis of small particles of crushed LC material were observed on a light microscope, as well as cells with vesicles that probably produce inflammatory mediators. The results of the TNF-α measurements revealed significant increase in TNF-α secretion in the case of LC material at both examined concentrations in unstimulated macrophages and LPS-stimulated macrophages at higher examined concentrations but also a significant decrease in the case of the SC material at both examined concentrations and both macrophage states. Unchangeable levels of nitrites in the case of unstimulated macrophages, a significant decrease in the case of LPS-stimulated macrophages for materials, and all concentrations except 100 µg/mL of SC material indicate that there are different mechanisms involved in the response of the macrophages to the examined materials and that this response is different in the case of LPS- and unstimulated macrophages. In a rare study dealing with the cellular inflammatory response induced by orthodontic adhesives, the in vitro inflammation behavior of primary human gingival fibroblasts was examined in contact with resin base and resin-modified glass ionomer base adhesives [39]. It has been shown that these adhesives decreased human gingival fibroblast survival and increased COX-2 mRNA and COX-2 protein expression in gingival fibroblasts [39]. When human neutrophils, isolated from peripheral blood, were exposed to the monomeric components of orthodontic bonding materials such as TEGDMA, Bis-GMA, UDMA, or TEGDMA in combination with Bis-GMA or UDMA, a significant increase in IL-8 release was noticed, compared to the unstimulated controls, while it did not affect apoptosis or necrosis of the exposed neutrophils [40]. The authors of this study also detected a synergistic pro-inflammatory effect of the combination of TEGDMA and Bis-GMA on neutrophils [40]. TEGMA is present in LC material tested in the present study in the mixture with Bis-GMA, and based on the literature data, the noticed increase in the level of pro-inflammatory cytokine TNF-α can be explained by the synergistic pro-inflammatory effect of these two components released from LC material.

Based on the results obtained in other studies, but also in the present study, the contact of the gingival tissue with orthodontics bonding materials should be avoided. The obtained results show that macrophage response is dependent on the macrophage inflammatory state and it differs in the case of self- and light-curing material. This indicates the need for careful application of orthodontic bonding materials and the thorough examination of the gingival condition prior to the placing of orthodontic appliances and potential anti-inflammatory treatments if necessary.

The limitations of the present study should be pointed out. In vitro cytotoxicity tests performed in the present study and examination of macrophages’ response, although widely used, cannot fully represent the cytotoxic properties of materials and inflammation occurrence in the oral cavity. It is known that the mucous membrane of the oral cavity is generally more resistant to toxic substances compared to the culture of cells due to layers of keratin that have a protective effect [41]. The present study was performed with the commonly used commercial cell lines of fibroblasts and macrophages in a monoculture cell system, which gives us the opportunity to analyze the effects on specific cell types that are the main cellular components of the oral tissue involved in the response to dental materials, and to study potential mechanisms of action at the cellular level. When interpreting the results obtained in the present study, we must bear in mind that due to the anatomy of the teeth, the short clinical crown and the technique of brackets’ gluing, the excess of material for bonding can often end up in the gingival sulcus. The epithelium of the gingival sulcus does not keratinize, so it is less resistant, and the material, by direct contact and through the release of soluble monomer molecules in gingival sulcus fluid, could then lead to the gingival inflammation. Based on obtained results and the knowledge gained in the present study, further research should be directed to the in vivo studies of the effects of the tested materials on the tissue level with the examination of the potential mechanisms of action, especially in long-term experiments.

## 5. Conclusions

According to the results obtained in the present study, the following conclusions can be made:

- There is a concentration- and time-dependent effect of the extracts of both examined orthodontic bonding materials on fibroblasts that suggests the differences in the dynamic and the amount of substances released from them;
- Self-curing bonding material proved to be more cytotoxic for fibroblasts in indirect culture systems in vitro in all examined time points compared to the light-curing material;
- There is a concentration- and time-dependent effect of the extracts, as well as the suspension of material particles, of both examined orthodontic bonding materials on macrophage metabolic activity;
- The macrophages’ response to examined materials in a direct cell culture system was dependent not only on the applied concentration of materials but also on the condition of macrophages and the presence of inflammatory agent.

All this suggests that prior to the selection and application of orthodontic bonding materials, all aspects should be considered regarding patient condition, the microenvironment in the oral cavity, potential inflammation of gingiva, etc. Further studies using in vivo models should be performed to examine the long-term effects of the use of different orthodontic bonding materials.

## Figures and Tables

**Table 1:** Properties of examined materials.

Material Properties	Non Mix Self-Cure Acrylic	Transbond™ XT Light Cure
Product information	ConTec Go! activator (Product code: 163–314) ConTec Go! adhesive (Product code: 163–321)	3M™ Unitek™ Transbond™ XT Primer (712-034) 3M™ Unitek™ Transbond™ XT Light Cure Adhesive (712-031, 712-036, 712-066)
Chemical composition *	Activator component: BISGMA dibenzoyl peroxide; benzoyl peroxide Adhesive component: BISGMA	Primer component: Bisphenol A Diglycidyl Ether Dimethacrylate (BISGMA) Triethylene Glycol Dimethacrylate (TEGDMA) 4-(Dimethylamino)-Benzeneethanol Adhesive component: Silane Treated Quartz Bisphenol A Diglycidyl Ether Dimethacrylate (BISGMA) Bisphenol A Dimethacrylate Silane Treated Silica Diphenyliodonium Hexafluorophosphate Triphenylantimony
Physical state (before polymerization)	Activator component: Liquid Adhesive component: Paste	Primer component: Liquid Adhesive component: Paste
Type of the material	Self-curing (SC)	Light-curing (LC)

* from the Safety Data Sheet (SDS) provided by manufacturers.

**Table 2:** Concentration of TNF-α (pg/mL) in macrophages’ cell culture media, measured by ELISA.

LPS Treatment	Concentration of Materials’ Suspension (µg/mL)				
	0	20		100	
		SC	LC	SC	LC
without LPS	49.37 ± 0.44	47.59 * ± 0.22	60.91 *** ± 0.22	42.26 *** ± 0.22	96.43 *** ± 0.38
		*p* < 0.001		*p* < 0.001	
with LPS	1204.27 ± 7.13	1161.52 ** ± 3.19	1168.88 ** ± 6.47	1012.72 *** ± 2.10	1261.10 ** ± 2.16
		/		*p* < 0.001	

(*) *p* < 0.05 (**) *p* < 0.01 (***) *p* < 0.001 compared to the corresponding control medium

**Table 3:** Concentration of nitrites (µM) in macrophage cell culture media, measured by Griess method.

LPS Treatment	Concentration of Materials’ Suspension (µg/mL)				
	0	20		100	
		SC	LC	SC	LC
without LPS	10.63 ± 0.52	10.04 ± 0.25	10.04 ± 0.25	10.38 ± 0.38	10.80 ± 1.01
with LPS	11.47 ± 0.88	9.46 * ± 0.38	8.79 * ± 0.25	17.43 *** ± 0.52	9.37 * ± 0.38

(*) *p* < 0.05 (***) *p* < 0.001 compared to the corresponding control medium

## Data Availability

All data are contained within the article.
